# Clustering of Directions Improves Goodness of Fit in Kinematic Data Collected in the Transverse Plane During Robot-Assisted Rehabilitation of Stroke Patients

**DOI:** 10.3389/frobt.2018.00057

**Published:** 2018-05-24

**Authors:** Ling Li, John Hartigan, Peter Peduzzi, Peter Guarino, Alexander T. Beed, Xiaotian Wu, Michael Wininger

**Affiliations:** ^1^Cooperative Studies Program, Department of Veterans Affairs, West Haven, CT, United States; ^2^Department of Biostatistics, Yale School of Public Health, New Haven, CT, United States; ^3^Department of Statistics, Yale University, New Haven, CT, United States; ^4^Statistical Center for HIV/AIDS Research and Prevention, Fred Hutchinson Cancer Research Center, Seattle, WA, United States; ^5^Department of Biostatistics, Brown University, Providence, RI, United States; ^6^Department of Rehabilitation Sciences, University of Hartford, West Hartford, CT, United States

**Keywords:** clustering, robot, rehabilitation, stroke, upper-limb

## Abstract

The kinematic character of hand trajectory in reaching tasks varies by movement direction. Often, direction is not included as a factor in the analysis of data collected during multi-directional reach tasks; consequently, this directionally insensitive model (DI) may be prone to type-II error due to unexplained variance. On the other hand, directionally specific models (DS) that account separately for each movement direction, may reduce statistical power by increasing the amount of data groupings. We propose a clustered-by-similarity (CS) in which movement directions with similar kinematic features are grouped together, maximizing model fit by decreasing unexplained variance while also decreasing uninformative sub-groupings. We tested model quality in measuring change over time in 10 kinematic features extracted from 72 chronic stroke patients participating in the VA-ROBOTICS trial, performing a targeted reaching task over 16 movement directions (8 targets, back- and forth from center) in the horizontal plane. Across 49 participants surviving a quality control sieve, 4.3 ± 1.1 (min: 3; max: 7) clusters were found among the 16 movement directions; clusters varied between participants. Among 49 participants, and averaged across 10 features, the better-fitting model for predicting change in features was found to be CS assessed by the Akaike Information criterion (61.6 ± 7.3%), versus DS (31.0 ± 7.8%) and DI (7.1 ± 7.1%). Confirmatory analysis via Extra Sum of Squares F-test showed the DS and CS models out-performed the DI model in head-to-head (pairwise) comparison in >85% of all specimens. Thus, we find overwhelming evidence that it is necessary to adjust for direction in the models of multi-directional movements, and that clustering kinematic data by feature similarly may yield the optimal configuration for this co-variate.

## Introduction

There is overwhelming evidence that the kinematics of voluntary movements vary depending on the direction of movement (Georgopoulos et al., 1988; Mushiake et al., 1991; Schwartz, 1993; Hewitt et al., 2011; Turnham et al., 2012). And yet, paradoxically, there is minimal precedence for accounting for this variability when analyzing data collected from multi-directional movement tasks. Here, we propose that including a factor term to account for movement direction can improve the goodness of model fit to multi-directional data sets; we test this paradigm in the setting of a ubiquitous line of inquiry, i.e., change in performance over time.

The cells comprising the primary motor cortex activate selectively in association with trajectory variables: each cell’s tuning centers on a preferred movement direction (Mushiake et al., 1991; Scott et al., 1997). This tuning varies from cell to cell (Georgopoulos et al., 1982, 1988), and the total population can co-vary with the hand trajectory (Schwartz, 1992, 1993; Scott et al., 1997). Many studies incorporating a multi-directional movement task via planar robots present data which evidence a substantial difference in the character of trajectories by direction (Yamamoto et al., 2006; Richardson et al., 2008; Brown et al., 2010; Hewitt et al., 2011; Turnham et al., 2012; Berniker et al., 2014; Muceli et al., 2014), an effect which persists in brain-injured patients (Shadmehr et al., 1998; Patton and Mussa-Ivaldi, 2004) and especially for stroke patients (Yarosh et al., 2004; Coderre et al., 2010; Scott and Dukelow, 2011). This observation is congruous with the notion that each person has preferred sectors of space through which arm movement is more proficient (Georgopoulos et al., 1986; Caminiti et al., 1990).

Given the thoroughness with which the neuromotor system has been shown to be directionally sensitive, established through studies of both cellular physiology as well as motor behavior, there is great need to develop robust strategies for adjusting for movement direction in analysis of kinematic data. The preponderance of studies make no mention of inclusion of any factor for direction whatsoever (Shadmehr et al., 1998; Rohrer et al., 2002; Daly et al., 2005; Finley et al., 2005; Hogan et al., 2006; Scheidt and Stoeckmann, 2007; Bosecker et al., 2010; Ganesh et al., 2014; Muceli et al., 2014; Sakamoto and Kondo, 2015); thus, we regard this approach as the “directionally insensitive” (DI) approach, where data from all movement directions are included in a single dataset with no directional co-variate term. Following the presumptive kinematic heterogeneity across multiple movement directions, we speculate that analytic approaches using the DI are more prone to error because of their substantial unexplained variance. A few studies take into account the different movement directions, typically by including a factor term for each direction (Scott et al., 1997; Yarosh et al., 2004; Maschke et al., 2004). While “directionally specific” (DS) models reduce the unexplained variance, it increases the number of groups in an ANOVA test. Such approaches require the estimation of more parameters than necessary, thereby reducing the amount of data available for hypothesis testing.

We conjecture that for a given patient, there may be some movement directions for which the kinematics are sufficiently similar so as to be combined with each other with minimal dilution. In this “clustered by similarity” (CS) model, we anticipate that by merging directions based on kinematic homogeneity, the unexplained variance will remain low, but the number of factors will decrease, thus increasing cell size, providing an intermediate alternative between the DI and the DS approaches. In this paper, we propose a method for merging movement directions based on features extracted from the kinematic record, and we compare the three approaches (DI, DS, and CS) by model fit.

## Methods

### Overview

In this study we examine improvement in the analysis of multi-directional kinematic data when the directions are included as factors in the analysis. Specifically, we test fit of the DI model versus two alternative approaches: (1) inclusion of a factor term for each distinct movement direction (DS), and (2) grouping of movement directions according to similarity among a set of standard kinematical features (CS). We perform this inquiry over kinematic data obtained from a cohort of stroke patients rehearsing targeted pointing movements with their affected limb.

### Study Population and Protocol

Data were obtained from participants in the VA-ROBOTICS study, a multi-center randomized and controlled clinical trial conducted at four VA medical centers between November 2006 and October 2008 (Lo et al., 2010). In the VA-ROBOTICS trial, patients with moderate-to-severe upper-limb impairment, at least 6 months removed from index stroke (i.e., the most recent stroke event, in case of recurrent stroke events), were randomly assigned to one of three treatment groups: Usual Care (UC), Robot-assisted Therapy (RT), or Intensive Comparison Therapy (ICT). Participants assigned to RT and ICT completed a comprehensive training regimen comprising 36 sessions over 12 weeks, driven by an adaptive robot (RT) or trained therapist (ICT); those assigned to UC did not receive prescribed training through the study, but were allowed to pursue collateral training independently from the study. Participants receiving ICT or UC were allowed to engage in robot-assisted rehabilitation after the completion of their follow-up; kinematic data were collected from the robot regardless of treatment assignment. Kinematic data were recorded from all participants interacting with the robot (Table 1).

**Table 1:** Baseline Characteristics of Study Participants.

Characteristics		Values
Sex – no. (%)	Male	75 (96.2)
	Female	3 (3.8)
Age - yr	—	64.3 ± 11.3
Treatment – no. (%)	Usual Care	18 (23.0)
	Robot-assisted Therapy	31 (39.7)
	Intensive Comparison Therapy	24 (30.7)
	No data	5 (6.4)
Stroke Type – no. (%)	Hemorrhagic	5 (6.4)
	Ischemic	67 (85.9)
	No data	6 (7.7)
Stroke Location	Anterior circulation (>1/3 of hemisphere)	13 (16.7)
	Small deep infarct	16 (20.5)
	Anterior circulation (<1/3 of hemisphere)	37 (47.4)
	Posterior circulation	12 (15.3)
Fugl-Meyer Score	—	19.8 ± 10.9

**Figure 1:**
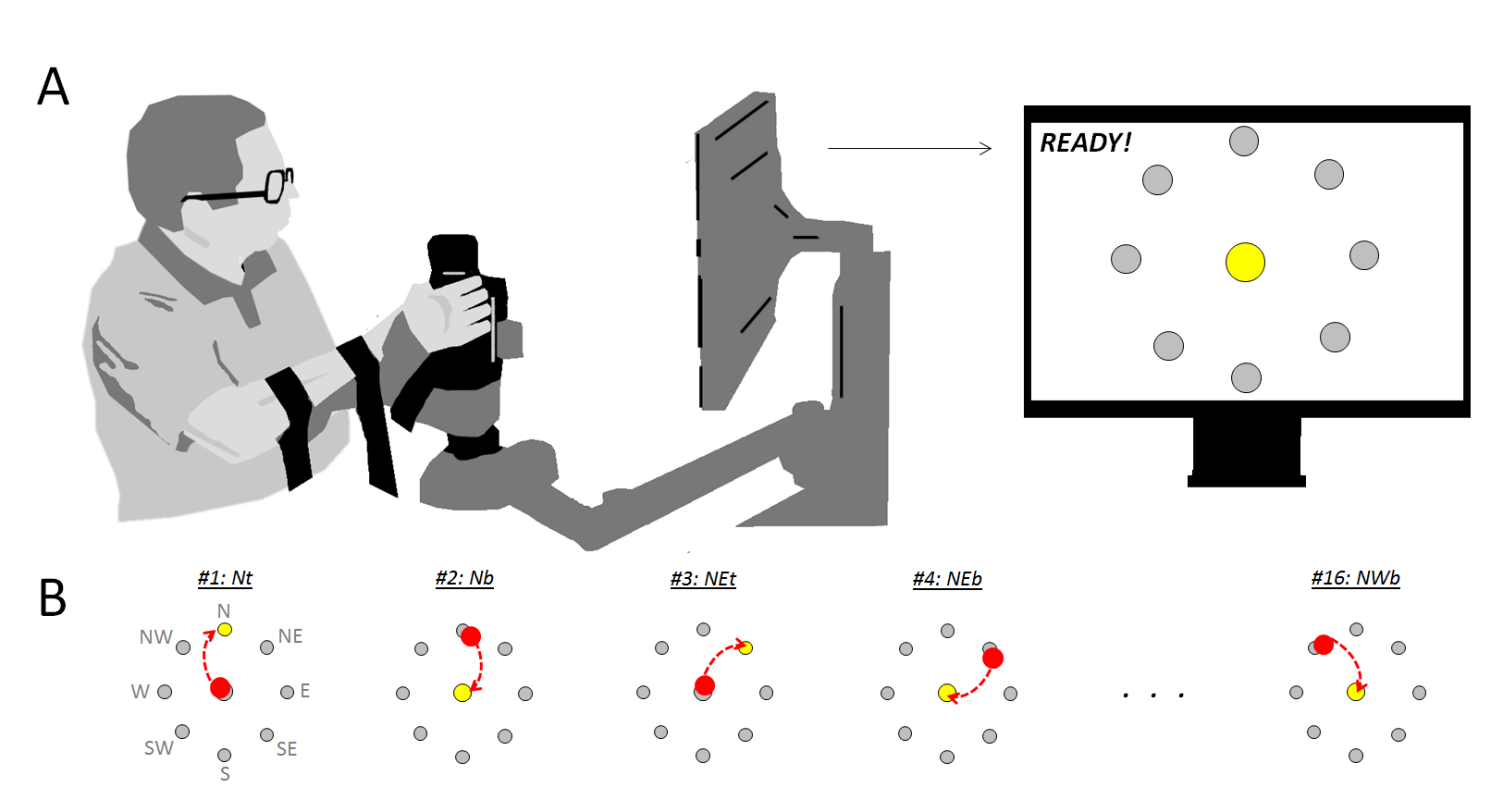
Visualization participant interacting with robot **(A)** and demonstration of target task **(B)**. Red dot shows current cursor location, dashed red line illustrates a trajectory toward the target location (yellow dot). Trajectory is shown in figure for clarity, but was not shown to participants as part of their visual feedback: only the targets and the cursor were shown.

The robot-assisted therapy was delivered via the In-Motion rehabilitation robotic system (Interactive Motion Technologies) (Aisen et al., 1997), which is specifically designed for clinical neurological applications. It delivers goal-directed, assisted upper-extremity movement, with an interactive computer-generated video program providing visual feedback to the patient. Participants engaged in a point-to-point movement task, comprising a series of up to 5 unassisted clockwise rotations through a circular target; there were 8 directional compass targets (N, NE, E, SE, S, SW, W, and NW) with both outward (from center to periphery, “toward”: “t”), and return (from periphery to center, “back”: “b”), yielding 16 total movement directions. North and South are oriented in the position of maximum and minimum outward reach, respectively, i.e., the positions farthest- and nearest position relative to the trunk. For an individual interfacing with the robot with their right-hand, East and West reflect the maximally lateral- and medial hand position, respectively (Figure 1, Top).

The robot pre-positioned the participant’s arm at the center target, and participants were expected to move toward the target in a single, smooth motion. When the participant was unable to complete a movement, the attending clinician could override the task by pressing the space bar, progressing to the next sequential target. Participants were instructed: “Your goal is to reach toward each of the red targets. If you are able to reach the respective targets, the robot will prompt you to move toward the next one. You will complete five cycles around the circle in a clockwise fashion. In the event you are unable to reach the target, I will pause the device and move your arm passively to the next start position. Tell me when you’re ready.”

Participants sat upright in a comfortable chair, and interacted with the robot through a manipulandum (Figure 1, Bottom). Pointing movements were made in the transverse plane, with a monitor at eye level displaying a cursor against a target field. Data consisted of instantaneous velocity in two-dimensions (left-right and fore-aft), sampled at 200 Hz. We smoothed these data bi-directionally with a 1st order low-pass Butterworth’s filter with 20 Hz cutoff; filter was re-applied after each differentiation.

The VA-ROBOTICS study was approved by the Institutional Review Board at each medical center and all study participants provided written informed consent.

### Data Conditioning

In order to avoid error associated with the robot’s automated data processing routine, serial files collected from the robot were concatenated and repartitioned according to movement reversals, yielding complete movement cycles reflecting a single point-to-point movement from start to finish (Beed et al., 2017). Movement cycles that were unusually short in duration were presumed to be measurement artefact due to clinician override because of participant inability to complete the movement. Accordingly, we censored movement cycles with duration 20 time samples (0.1 s) or with variance in velocity = 0, corresponding with a spuriously brief or stationary data sample, respectively. All data conditioning activities were performed on the comprehensive dataset, i.e., all participants, all days at once.

### Feature Extraction

In order to adequately capture the kinematic character of these data, we extracted 10 features that have been shown to be labile to movement direction (de Rugy et al., 2013; Hamel-Paquet, 2006; Hewitt et al., 2011; Maschke, 2003; Muceli et al., 2014). In addition to basic movement parameters of amplitude, duration, average- and peak velocity, average- and peak acceleration, we calculated four descriptors related to movement smoothness including the number of peaks in the velocity trace (Fetters and Todd, 1987; Michaelsen et al., 2006; Thelen et al., 1993), the tent metric i.e., the ratio of the area under the convex hull fitted to the velocity trace versus the area under the velocity trace itself (Rohrer et al., 2002), and two formulations of jerk (Hogan and Sternad, 2009; Rohrer et al., 2002). Where jerk-based metrics have been shown to be especially sensitive to basic movement parameters (Wininger et al., 2009, 2012), we tested one formulation witha widely analyzed behavior $j_{1}=\frac{1}{v_{max}\cdot \left(t_{2}-t_{1}\right)}\int_{t_{1}}^{t_{2}}\left|\dddot{x}\right|dt$, where *t*_1_ and *t*_2_ are the start-time and cessation time of the movement, *v*_max_ is the maximum velocity, and $\dddot{x}$ is the third time derivative of position; *j*_1_ has dimensions *T*^−2^ (Rohrer et al., 2002), and one recently-proposed measure designed to be robust to these parameters $j_{2}=\frac{{\left(t_{2}-t_{1}\right)}^{5}}{A^{2}}\int_{t_{1}}^{t_{2}}{\dddot{x}}^{2}dt$, where *A* is the total movement distance; *j*_2_ is non-dimensionalized (Hogan and Sternad, 2009).

Features were tested for normality via Shapiro-Wilk test; features found to be non-normal were log-transformed. Within each feature, outliers were identified as observations more than 3 standard deviations from the mean across all participants; movement cycles yielding one or more outlier value were discarded.

Our main objective in this study was to establish patterns of similarity or difference between movement cycles, based on their performance, i.e., characteristics of their trajectory. Because these features are expected to be highly collinear (Wininger et al., 2009, 2012), feature set dimensionality was reduced via Principal Components Analysis (PCA) over the entire dataset, i.e., all participants all days. A minimum threshold was established a priori at 80%: the minimum number of PCs accounting for greater than 80% of the variance would be retained for analysis.

### Cluster Identification

In order to test for directions with kinematic homology, participant-level data were assessed via a greedy pair-wise optimization of the Mahalanobis Distance

 (1) $$D_{ij}={\left({\vec{x}}_{i}-{\vec{x}}_{j}\right)}^{T}{\left(\frac{S_{i}}{n_{i}}+\frac{S_{j}}{n_{j}}\right)}^{-1}\left({\vec{x}}_{i}-{\vec{x}}_{j}\right)$$

where x_i_ and x_j_ are the means of the PC-transformed features of direction *i* and *j* in *k* dimensions, where *k* = the number of re-combined features surviving PCA threshold; *n*_i_ and *n*_j_ are the sample sizes (number of movements cycles in each direction), and *S*_i_ and *S*_j_ the covariance matrices of the PC-transformed features for each direction. We obtain the p-value of each *D*_ij_ due to the fact that *D*_ij_ is approximately Chi-square distributed with degree of freedom k, under the null hypothesis that the PC-transformed features in direction *i* are sampled from the same multi-variate Gaussian distribution as the PC-transformed features from direction *j*, equal to the number of dimensions mentioned above. The null hypothesis is: *D*_ij_ is not significantly different from zero, indicating the two directions are similar to each other based on Mahalanobis Distance and should be merged together. In the first iteration, this distance was computed for all 1 ≤ *i* ≤ 15 and *i* + 1 < j ≤ 16 (120 total comparisons); if the two directions with the minimum *D*_ij_ yielded a non-significant difference (*p*_max(Dij) _> 0.05), the directions were merged into a new cluster (yielding 15 clusters total), and the process re-iterated for all 1 ≤ *i* ≤ 14 and *i* + 1 < j ≤ 15 (105 total remaining comparisons), and so-on. For each round of mergers, the Mahalanobis threshold was adjusted for multiple comparisons via the Bonferroni correction. This process continued until no pair-wise D yielded significance.

The Mahalanobis Distance requires *S*_i_ and *S*_j_ to be invertible, meaning that covariance matrix should be full-rank. Thus, the number of observations for each task must be equal to or greater than the number of PCs. To ensure viability, it was decided *a priori* to merge data over all days; participants with insufficient data were removed from the analysis stream.

### Statistical Models

For each participant, three models were tested:

Directionally Insensitive model (DI)

 (2)  $$fi=\;\beta_{0}+\beta_{1}*Day$$

Directionally Specific model (DS)

 (3) $$fi=\;\beta_{0}+\beta_{1}*Day+\sum_{j=2}^{16}\beta_{2j}*Dir_{j}+\sum_{j=2}^{16}\beta_{3j}*\left(Day*Dir_{j}\right)$$

Clustered by Similarity model (CS)

 (4) $$fi=\;\beta_{0}+\beta_{1}*Day+\sum_{j=2}^{J}\beta_{2j}*Clust_{j}+\sum_{j=2}^{J}\beta_{3j}*\left(Day*Clust_{j}\right)$$

where *f*_i_ is the *i*^th^ feature, 1 ≤ *i* ≤ 10, and *J* is the total number of clusters for the participant; β_0_ is the intercept and *β*_1,2,3_ are the regression slope coefficients. *Day* is a factor variable related to the date of the visit (for assessing change across multiple visits), *Dir* indicates one of 16 directions, and *Clust* indicates one of J clusters. The DI represents the prevalent statistical model for showing change in a variable over a period of observation. The DS is designed to increase the proportion of explained variance of the DI by accounting for the 16 movement directions, but at the cost of reduced sample size in the model comparisons. The CS putatively decreases the complexity of the DS by consolidating directions where the Mahalanobis Distance is minimized, thus decreasing the number of comparisons thereby increasing comparison sample size. We hypothesized that both the DS and CS will improve model fit versus the DI. These equations are intended to variously extend the models presented in the study’s previous outcomes assessments (Lo et al., 2010; Wu et al., 2016). We are unaware of these specific models, particularly DS or CS, having been used elsewhere previously.

### Model Quality

Firstly, a hierarchy of model quality was established via Akaike Information Criterion: AIC = 2 k – 2 ln [L], where *L* is the maximized likelihood, and k is the number of terms in the model. There is no rigorous test of model optimality for comparing heterogenous multi-level models, but the AIC is a useful heuristic (Akaike, 1981; Sin and White, 1996). For each participant, for each feature, we measured the frequency of AIC minimization by the three models.

Additionally, for each of the three possible outcomes (AIC minimized through DI, DS, or CS), the difference between the AIC values for the best-performing model was calculated for the two remaining models. Through this, we seek to measure the relative improvement of the optimum model over the two alternatives.

We observe that the DI can be considered nested within the CS, which in turn can be considered nested within the DS. Thus, model quality can be tested in these two pair-wise comparisons via the Extra Sum of Squares F test

(5) $$F=\frac{\frac{SSE_{Model1}-SSE_{Model2}}{df(SSE_{Model1})-df(SSE_{Model2})}}{\frac{SSE_{Model2}}{df(SSE_{Model2})}}$$

where this test statistic has a F[df(SSE_Model1_)−df(SSE_Model2_), SSE_Model2_] distribution, and Model1 corresponds to the model nested within Model2. Null hypothesis is the simpler model (Model1) fits adequately on the data (*p*-value > 0.05). For each participant, for each feature, we measured the frequency of significant test results in three comparisons: DI versus CS, DI versus DS, and CS versus DS.

## Results

### Descriptive Statistics

Data were collected from all participants engaging with the robot. In total 13,977 movement cycles were captured over 202 participant days. All movement cycles were found to be of adequate data quality to support analysis; 996 movement cycles were removed as outliers and 19 participants (1,134 movement cycles) were discarded due to a lack of a second recorded session with the robot. Among the surviving movement cycles, we observe that their basic kinematic parameters appear to commensurate with a moderately-impaired cohort (Table 2).

**Table 2:** Basic kinematic parameters; F = Feature.

	Parameter value: Average ± SD
F1_Amplitude (meters, m)	0.81 ± 0.017
F2_Duration (seconds, s)	0.58 ± 0.135
F3_AverageVelocity (m/s)	0.33 ± 0.11
F4_PeakVelocity (m/s)	0.99 ± 0.32
F5_AverageAcceleration (m/s^2^)	1.135 ± 0.39
F6_PeakAcceleration (m/s^2^)	3.84 ± 0.14
F7_JerkVelNormalized (1/s^2^)	2.0 ± 0.28
F8_JerkNonDimensionalized (n/a)	5.3 ± 1.10
F9_VelocityPeaks (n/a)	1.1 ± 0.19
F10_VelocityTent (m)	0.13 ± 0.01

We note that in a two-way ANOVA, movement direction and the interaction term between movement direction and participant ID yielded significant *p*-values for all 10 features (results not shown). This supports the assumption of a sensitivity of kinematic performance to movement direction.

### Principal Components

Most participants (35 of 49) required two PCs to reach the 80% threshold; the remainder (14 of 49) needed three. Subsequently ten participants (677 movement cycles) yielded datasets with one or more movement directions containing insufficient data to execute clustering, and were removed from the analysis stream. The final dataset comprised 11,119 movement cycles performed by 49 participants captured over 154 participant days. A correlation matrix for these features is shown in Table 3.

**Table 3:** Correlation matrix of extracted features; F = Feature.

	F1	F2	F3	F4	F5	F6	F7	F8	F9	F10
F1_Amplitude	1	0.58	0.19	0.43	0.39	0.44	0.29	0.54	0.40	0.69
F2_Duration	0.58	1	−0.62	0.16	−0.26	0.22	−0.03	0.87	0.93	0.27
F3_AverageVelocity	0.19	−0.62	1	0.22	0.71	0.16	0.30	−0.52	−0.68	0.26
F4_PeakVelocity	0.43	0.16	0.22	1	0.67	0.95	0.79	0.53	0.10	0.77
F5_AverageAcceleration	0.39	−0.26	0.71	0.67	1	0.66	0.77	0.06	−0.35	0.66
F6_PeakAcceleration	0.44	0.22	0.16	0.95	0.66	1	0.89	0.62	0.16	0.77
F7_JerkVelNormalized	0.29	−0.03	0.30	0.79	0.77	0.89	1	0.43	−0.05	0.65
F8_JerkNonDimensionalized	0.54	0.87	−0.52	0.53	0.06	0.62	0.43	1	0.83	0.51
F9_VelocityPeaks	0.40	0.93	−0.68	0.10	−0.35	0.16	−0.05	0.83	1	0.17
F10_VelocityTent	0.69	0.27	0.26	0.77	0.66	0.77	0.65	0.51	0.17	1

### Clustering

**Figure 2:**
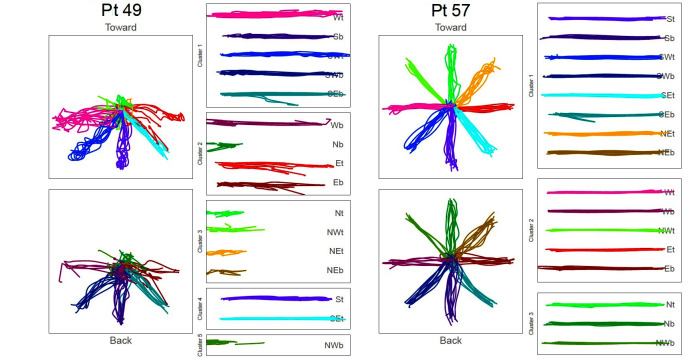
Kinematic data collected from two stroke patients reflecting movement of the robot manipulandum in transverse plane. Data shown ensemble as recorded from robot (*Left Panels*), as well as rotated and grouped by cluster (*Right Panels*).

Across the remaining participants, the data clustered into 4.3 ± 1.1 groupings (min: 3; max: 7). Among the single-most populated cluster, the number of movement directions contained therein was variable with a mean ± SD deviation of 7.5 ± 2.2 directions (min: 4; max: 14). Figure 2 shows two exemplars of participant data with clustering results.

In Figure 2, the star-shaped portraits show kinematic data super-imposed in the plane of movement in *toward* movements (top) and back (bottom) directions. The columns showing break-out boxes with horizontal traces are the kinematic data, grouped by cluster, and rotated to left-to-right orientation for visualization. Participant #49 exhibits severely impaired movement; Participant #57 shows proficient movement. While space limitations preclude an extended analysis of the differences and similarities of the clusters across participants, we report that there is no one movement direction consistently grouped within the largest cluster (Table 4).

**Table 4:** Representation of Directions among participants' largest clusters.

	N	NE	E	SE	S	SW	W	NW
t	22.4	44.9	49.0	53.1	38.8	55.1	59.1	32.7
b	51.0	51.0	34.7	53.1	53.1	53.1	53.1	46.9

Proportion of participants for whom the Direction was contained within the participant's largest cluster (percent). b = movement back from target; t = movement towards target.

### Model Quality

Table 5 presents the frequency of model optimization and the margin of model improvement for each feature at the *p* = 0.05 threshold, and for all features combined for all three thresholds.

**Table 5:** Model Quality Results: Frequency of model superiority, and margin of improvement; F = Feature.

	DI optimal			DS optimal			CS optimal		
	Freq	AIC_DI_–AIC_DS_	AIC_DI_– AIC_CS_	Freq	AIC_DS_–AIC_DI_	AIC_DS_–AIC_CS_	Freq	AIC_CS_–AIC_DI_	AIC_CS_–AIC_DS_
	%	M (IQR)	M(IQR)	%	M (IQR)	M (IQR)	%	M (IQR)	M (IQR)
F1	22.4	−32 (−30–−16)	−8 (−9–−6)	22.4	−64 (−77–−54)	−28 (−35–−22)	55.1	−21 (−37–−6)	−20 (−29–−8)
F2	0	—	—	32.7	−56 (−94–−46)	−16 (−26–−6)	67.3	−36 (−63–−24)	−22 (−34–−11)
F3	0	—	—	44.9	−79 (−117–−59)	−14 (−34–−8)	53.1	−35 (−61–−28)	−17 (−24–−10)
F4	8.2	—	—	30.6	−77 (−119–−41)	−22 (−30–−11)	61.2	−34 (−62–−12)	−22 (−31–−11)
F5	4.1	—	—	26.5	−77 (−93–−31)	−20 (−41–−14)	69.4	−32 (−45–−19)	−23 (−29–−14)
F6	10.2	—	—	22.4	−54 (−79–−31)	−16 (−39–−13)	67.3	−29 (−51–−10)	−26 (−36–−13)
F7	10.2	—	—	32.7	−46 (−59–−34)	−20 (−29–−6)	57.1	−22 (−47–−14)	−27 (−31–−10)
F8	0	—	—	40.8	−78 (−123–−54)	−16 (−36–−6)	59.2	−62 (−96–−24)	−21 (−31–−11)
F9	4.1	—	—	22.4	−62 (−106–−43)	−11 (−19–−4)	73.5	−35 (−61–−30)	−17 (−31–−11)
F10	12.2	−38 (−44–−34)	−7 (−8–−4)	34.7	−37 (−78–−25)	−14 (−31–−9)	53.1	−22 (−50–−11)	−17 (−27–−9)
All	7.1 ± 7.1	−32 (−40–−18)	−7 (−40–−3)	31.0 ± 7.8	−63 (−94–−37)	−17 (−94–−8)	61.6 ± 7.3	−33 (−61–−17)	−20 (−61–−11)

AIC = Akaike Information Criteria; DI = Directionally Insensitive model, DS = Directionally Sensitive model, CS = Clustered by Similarity model. Frequency presented as proportion of all models in percent; AIC comparison presented as median difference with inter-quartile range computed within the subset of participants for whom the model yielded optimal AIC. Bottom row presents average (± SD) across all 10 features.

In particular, we note that the Directionally Insensitive model is best only 7% of the time (average across all features), and when the DI is best, it provides only a modest improvement over the clustered model, decreasing AIC by only 7.0 points versus CS (IQR 3.2–40.4 points decrease). The DS was the optimum model 31% of the time, although we note that in those cases where DS was the best model, its impact on AIC minimization was substantial: 62.8 points reduction in AIC versus CS (IQR: 36.7–94.3 points decrease). The CS yielded the superior model 62% of the time, with moderate improvement over the other two models: −33.1 (−61.0–−17.1) versus DI and −20.2 (−61.0–−11.0) versus DS.

Comparison via the F-test showed similar results (Table 6). The DS was consistently superior to the DI: range across 10 features, the F-test yielded *p* < 0.05 in at least 75% of samples, and as much as 96% (average: 86.7 ± 7.3%). Similarly, the CS proved superior to DI: range 78 to 100% (average: 91.2 ± 7.9%). We note that the margin of improvement of using DS versus CS was moderate: 36 to 67% (average: 49.6 ± 7.5%).

**Table 6:** F test results between different model comparisons for each feature; within table: F = Feature.

	DS vs. DI	CS vs. DI	CS vs. DS
	Freq (*p* ≤ 0.05)	Freq (*p* ≤ 0.05)	Freq (*p* ≤ 0.05)
F1_Amplitude	77.6	75.5	44.9
F2_Duration	95.9	100	49.0
F3_AverageVelocity	93.9	98.0	65.3
F4_PeakVelocity	83.7	89.8	46.9
F5_AverageAcceleration	89.8	93.9	44.9
F6_PeakAcceleration	79.6	89.8	36.7
F7_JerkVelNormalized	77.6	85.7	51.0
F8_JerkNonDim.	95.9	100	53.1
F9_VelocityPeaks	89.8	95.9	49.0
F10_VelocityTent	83.7	83.7	55.1
All features	86.7 ± 7.3	91.2 ± 7.9	49.6 ± 7.5

DI = Directionally Insensitive model, DS = Directionally Sensitive model, CS = Clustered by Similarity model. Frequency presented as raw count with proportion of all models in percent: N (%). Bottom row presents average (± SD) across all 10 features.

## Discussion

### Study Validity

In this study, we test whether inclusion of a factor for movement direction increases model fit in the context of a simplistic, but common hypothesis test, i.e., change in a kinematical feature over time. Testing this method with data collected from an impaired cohort over an extended time period (stroke patients from the VA-ROBOTICS study, with 217 ± 172 days between first- and final measurements) is strategic in that there is a greater likelihood that some participants will show non-trivial change in these features over time, providing greater opportunity to capture an association between response and predictor.

While we considered assessing kinematic similarity based on the raw trajectory, we decided in the end to predicate our clustering on features extracted from the trajectory. While recognize that some will consider clustering-by-trajectory as a more direct approach, we believe clustering within the feature space to be a reasonable surrogate for trajectory, and caution that comparison of trajectories is a complex enterprise: patients with neuromotor dysfunction can move slowly or rapidly, and sometimes both slowly and rapidly in the same data sample. In moderately-impaired populations, movement arrest can be particularly prevalent (Beppu et al., 1984; Rohrer et al., 2002), and can confound attempts to obtain meaningful waveform correlations (Wininger et al., 2009).

And while not an explicit objective of this study, we note that this study partly replicates the findings of others, published in this journal who demonstrated that there is substantial efficiency to be gained –with minimal loss in accuracy– in reducing model complexity in kinematic analysis of human upper-limb movement in robotic systems (Averta et al., 2017).

### Interpretations

The paradigm of adding a new factor to an analysis changes both statistical power, and model interpretation. Given the vastness of existing studies where planar robots are used to capture kinematics in multi-dimensional movement, it is impractical to attempt to address the implications of a DI approach with specificity. Broadly speaking, one prominent impact of the new CS approach is the opportunity to re-analyze data presented in previous studies: non-significant results may become significant due to the identification of homogeneity across multiple movement directions, and significant results could benefit from increased effect size. Equivalently, confirming negative findings through the incorporation of CS would add value.

There is inherent complexity in utilizing two assays (minimization of AIC versus frequency of significant F-tests). We assert the value in this pluralistic approach: two distinct analyses provide mutual validation in the absence of a gold-standard approach. At the same time, we recognize that the results, while largely consistent, diverge somewhat in the assessment of DS versus CS. Per AIC, CS is the better model 61% of the time (Table 5), per F-test, CS fails to yield significantly better results in more than half the cases (50.4%, Table 6). We view these as compatible results: the discrepancy is small (approximately 10% absolute difference), and is likely attributable to the disparate nature of the measures: AIC minimization is arithmetically based, where F-test involves transformation and thresholding. Taken together, we believe that CS does provide non-trivial improvement versus DS, but additional work may be helpful in illuminating the true magnitude of benefit.

### Application

There is progressively greater evidence that rehabilitation robots are most efficacious when employing a cooperative control strategy versus passive support (Israel et al., 2006; Ziherl et al., 2010; Balasubramanian et al., 2012; Casadio and Sanguineti, 2012; Van der Loos et al., 2016). And it is generally preferred that a rehabilitation robot offer assistance in a way that reflects the level of impairment (Novak and Riener, 2015), which can vary across the workspace: movements in some directions are more proficient than others. Physical therapists are experts at identifying areas of weakness, and prioritizing those areas for focused rehabilitation (Sahrmann, 1988). Rehabilitation robots provide obvious advantages: precision placement and support, durability, and analytical sophistication. However, because the performance measures available for quantifying motor skill are many and varied, and collinearities are common (Table 3), there remain substantial opportunities to streamlining robot measurement and control strategies so that they are efficient and more human-like. We note that the interest in bridging the gap between rehabilitation robots and their human counterparts extends to identification of kinematical factors underlying the clinical functional scores (Bosecker et al., 2010; Scott and Dukelow, 2011; van Dokkum et al., 2014). We anticipate that our approach may provide new inroads towards understanding the subtle, intuitive approach of the human therapist in assessing motor skill.

### Limitations

In terms of study design, this work can be considered generalizable in the sense that we analyzed data from patients in active rehabilitation programs, as well as those with no ongoing training. This study is limited by participant demography: we included primarily male patients, with mild- to moderate impairment due to stroke, an older population with substantial co-morbid burden. Furthermore, space limitations necessarily narrow the scope of this paper to purely technical matters; there is limited opportunity for meaningful clinical or physiological inquiry.

Methodologically, this approach faces some limitations. Primarily is that due to sample size. In order to prevent singularity, there must be at least *N* + 1 samples in each movement direction, where N is the dimensionality of the feature space. We accommodated this restriction by reducing feature space dimensionality through principal components analysis. PCs reflect a weighted sum of the variables, accounting for a progressively smaller amount of variability among the observed data. Increasing to a higher dimensionality, while preferable for clustering robustness, would have meant the removal of more participants’ data. We note that while many participants had ample data in most movement directions, many participants had difficulty in just one or two movement directions; this approach requires adequate samples in all movement directions. As a consequence of the use of principal components, the clustering is performed in a 2-D or 3-D space described by a linear re-combination of features, as opposed to the full 10-D space created by the raw features. On the other hand, our results (first two PCs yielded >80% of the variance) suggests the sufficiency of a low-dimensional transformed feature space, and the additional sample size gained through use of PCA evidences its value. Moreover, PCA provides excellent protection against feature collinearity: there is a reduced burden in feature design and selection when the features are re-combined in a way that maximizes their combined proportional variance.

Analytically, we note that this study is limited by the lack of clear tools for concise measurement of model goodness of fit. In order to convincingly test the three models, we analyzed in two ways: comparison via AIC, and via the Extra Sum of Squares F-test. We anticipate that in many cases, both the AIC and the F-test will be supported, but we acknowledge that the AIC is not universally considered a robust approach for model comparison, and the F-test does not easily avail to comparison beyond the binary assessment of above- or below the pre-defined significance threshold.

The Mahalanbois distance was chosen as the preferred approach for clustering out of expeditiousness. So-called greedy methods such as serial re-grouping based on minimum-distance criterion applied to a small number of candidate models, are not comprehensive, and therefore we cannot be certain that the results reported here reflect the true utility of the CS. A fully complete test of the CS would require assessment of model fit in all possible regression models. However, this poses an intractably large calculation: for the 1-cluster regression, there is a single candidate model (this is the DI); for the 2-cluster regression, there are $\sum_{i=0}^{15}C_{i}^{15}$ = 32,768 ways to divide 16 movement directions into two clusters; for the 3-cluster regression, there are $C_{2}^{16}\cdot \sum_{i=0}^{14}\left(C_{i}^{14}\cdot \left(\sum_{j=0}^{14-i}C_{j}^{14-i}\right)\right)$ = 573,956,280 ways to divide 16 movement directions into 3 clusters, and so on. A complete search across all candidate models would require many billions of calculations per participant-feature; given our sample size (n = 72 participants x 10 features), a full search is simply not feasible. Nevertheless, while our greedy model cannot be interpreted as exhaustive, the CS model showed consistent superiority versus the DI and DS, and could only be enhanced by more extensive search. Summarily, we assert that our incorporation of the Mahalanobis Distance as a heuristic for clustering directional data –while not exhaustive– was efficient and effective, and yielded results which strongly evidence the superiority of the CS.

### Extension and Future Work

The present study is the first, known to the authors, to directly test the impact of movement direction as a co-variate in the kinematical analysis. Furthermore, ours is the first study to identify the tradeoff between explained variance and cell size in the two extant approaches (DI versus DS), and to propose an alternative, i.e., CS. As a result, this study has generated new knowledge, not only for methodological rigor, but for the study of human motor behavior: we find that the 16 movement directions cluster naturally into approximately 4 groups of kinematic similarity in mild- to moderately impaired chronic stroke patients (*p* < 0.05 threshold).

In the interest of brevity and clarity we defer focused analysis of the trajectory clustering. However, our preliminary inquiry reveals an intriguing cluster patterning. Movement directions were noted for number of times they clustered with each of the 15 other movement directions (Table 7). We highlight those cells with a large number of shared clusters (*n* ≥ 20), and note the highly diagonal orientation of these co-clusters within the matrix. From this, we identify the following approximate clusters in the all-participants view: {NWt, Nt, NEt}, {NWb, Nb, NEb}, {SEb, Sb, SWb}. In particular, we observe: (1) none of the backwards directions are associated with towards directions, (2) both towards and back directions have distinct groupings of North and South.

**Table 7:** Co-clustered movement directions across participants.

	NWt	Nt	NEt	Et	SEt	St	SWt	Wt	NWb	Nb	NEb	Eb	SEb	Sb	SWb	Wb
NWt	100	65	49	16	20	20	24	31	14	18	22	10	10	18	16	10
Nt	65	100	53	14	20	27	10	20	12	20	10	8	0	6	8	6
NEt	49	53	100	27	29	27	22	31	10	35	24	14	20	31	16	14
Et	16	14	27	100	33	18	29	43	20	24	20	39	27	22	20	35
SEt	20	20	29	33	100	53	47	20	18	29	18	22	20	24	16	18
St	20	27	27	18	53	100	35	22	14	20	16	12	16	27	22	20
SWt	24	10	22	29	47	35	100	37	20	22	29	27	29	31	29	18
Wt	31	20	31	43	20	22	37	100	22	18	24	33	33	31	35	39
NWb	14	12	10	20	18	14	20	22	100	45	43	35	39	24	24	45
Nb	18	20	35	24	29	20	22	18	45	100	49	20	37	29	22	35
NEb	22	10	24	20	18	16	29	24	43	49	100	41	35	33	33	41
Eb	10	8	14	39	22	12	27	33	35	20	41	100	33	18	24	41
SEb	10	0	20	27	20	16	29	33	39	37	35	33	100	55	45	47
Sb	18	6	31	22	24	27	31	31	24	29	33	18	55	100	45	31
SWb	16	8	16	20	16	22	29	35	24	22	33	24	45	45	100	43
Wb	10	6	14	35	18	20	18	39	45	35	41	41	47	31	43	100

Cells report percentage of participants for whom two directions appear in the same cluster. Average off-diagonal element: 26.4 ± 12.0; elements greater than *n* = 20 (40%) of participants yielding co-clusterings highlighted.

Replication will be required in order to measure the clustering in other populations, including normative data on healthy controls. Additional exploration will give insight into whether these clusters are consistent across patients, whether the movement directions in some clusters are more sensitive to training than others, and whether a cluster-based approach will provide badly needed leverage in the ongoing research into treatment response (Lo et al., 2010), and the association between kinematics and clinical-based performance measures (Bosecker et al., 2010).

## Disclaimer

Opinions herein are those of the individual authors and the contents do not represent the views of the Department of Veterans Affairs of the United States Government.

## Ethics Statement

The study protocol was approved by the institutional review boards at each participating site and the human rights committee at the coordinating center, listed as follows: VA Maryland Health Care System (Baltimore), North Florida/South Georgia Veterans Health System (Gainesville), VA Puget Sound Health Care System (Seattle), and VA Connecticut Healthcare System (West Haven). All participants gave written informed consent prior to study participation. The study was registered on ClinicalTrials.gov (ClinicalTrials.gov identifier, NCT 00372411).

## Author Contributions

This secondary study was initially conceived and conducted by LL, JH, XW, and MW. Original trial design and operations was overseen by PG and PP. Data conditioning and code review was performed by AB and MW. All authors were involved in the drafting and approving of this manuscript.

## Conflict of Interest Statement

The authors declare that the research was conducted in the absence of any commercial or financial relationships that could be construed as a potential conflict of interest.
